# Increasing Accessible Active Site Density of Non-Precious Metal Oxygen Reduction Reaction Catalysts through Ionic Liquid Modification

**DOI:** 10.1021/acsami.2c21441

**Published:** 2023-04-06

**Authors:** Gui-Rong Zhang, Cong Yong, Liu-Liu Shen, Hui Yu, Kai Brunnengräber, Timo Imhof, Donghai Mei, Bastian J. M. Etzold

**Affiliations:** † School of Chemical Engineering and Technology, Tiangong University, Binshuixi Road 399, Tianjin 300387, China; ‡ Department of Chemistry, Ernst-Berl-Institut für Technische und Makromolekulare Chemie, Technical University of Darmstadt, Alarich-Weiss-Street 8, Darmstadt 64287, Germany; § School of Chemistry, Tiangong University, Binshuixi Road 399, Tianjin 300387, China; ∥ School of Environmental Science and Engineering, Tiangong University, Binshuixi Road 399, Tianjin 300387, China

**Keywords:** non-precious metal catalysts, oxygen reduction reaction, ionic liquids, ZIF-derived carbon, electrochemically active surface area

## Abstract

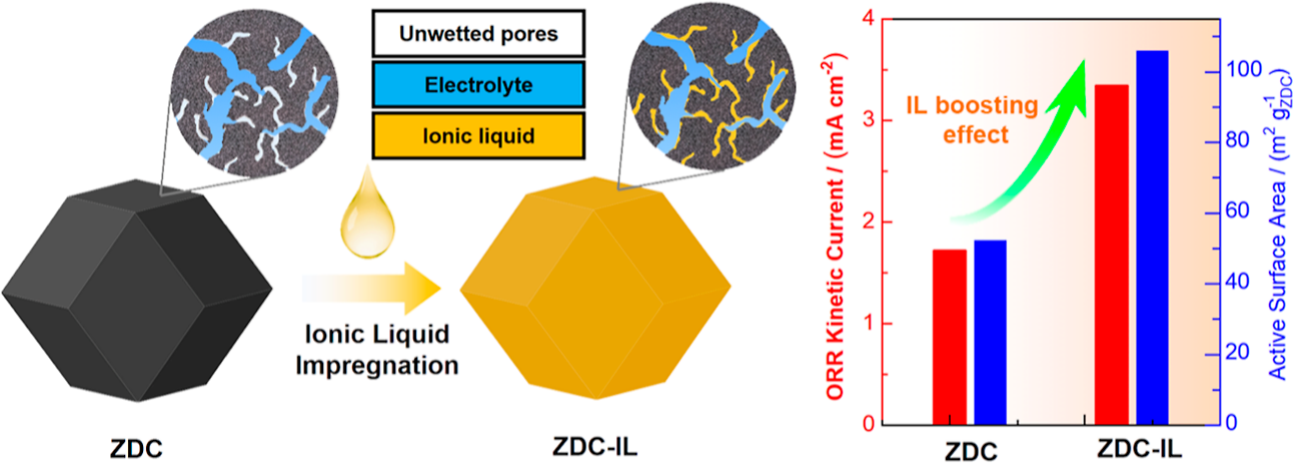

Non-precious metal catalysts show great promise to replace the state-of-the-art Pt-based catalysts for catalyzing the oxygen reduction reaction (ORR), while their catalytic activity still needs to be greatly improved before their broad-based application. Here, we report a facile approach to improving the performance of zeolitic imidazolate framework-derived carbon (ZDC) toward the ORR by incorporating a small amount of ionic liquid (IL). The IL would preferentially fill the micropores of ZDC and greatly enhance the utilization of the active sites within the micropores, which are initially not accessible due to insufficient surface wetting. It is also disclosed that the ORR activity in terms of kinetic current at 0.85 V depends on the loading amount of the IL, and the maximum activity is obtained at a mass ratio of IL to ZDC at 1.2. The optimum stems from the counterbalance between the enhanced utilization of the active sites within the micropores and the retarded diffusion of the reactants within the IL phase due to its high viscosity.

## 1 Introduction

The oxygen reduction reaction (ORR) represents a cornerstone for many key energy conversion technologies such as fuel cells and metal–air batteries, which hold great potential as clean power sources for portable and transportation applications.^1,2^ However, the large-scale deployment of these clean energy conversion devices/technologies is limited by the slow kinetics of the ORR, which brings down the energy efficiency of these devices.^3−5^ In view of this, intensive efforts have been made to accelerate the ORR kinetics, mainly by constructing innovative electrocatalysts, among which platinum (Pt)-based materials are usually regarded as the best-performing catalysts.^6^ Nevertheless, Pt-based ORR catalysts suffer from high cost due to the scarcity of Pt, and this challenge will be escalated when facing the commercialization of low-temperature fuel cells or metal–air batteries.^7^

The cost issue of Pt has stimulated the development of ORR catalysts free from Pt or any other precious metals, i.e., non-precious metal catalysts (NPMCs), which represents an exciting research field with the potential to meet the cost requirements for large-scale applications. The pioneering work can date back to 1964, when Jasinski reported for the first time that cobalt phthalocyanine was an active ORR catalyst in the KOH electrolyte.^8^ However, the breakthrough in ORR performance was not achieved until the last 2 decades, when a variety of high-performing NPMCs start emerging, which were typically prepared via the pyrolysis of the compounds or mixtures containing nitrogen and carbon sources with/without transitional metals (e.g., Fe, Co, and Mn).^9−11^ Nevertheless, the performance of NPMCs is still far from satisfactory when compared to the state-of-the-art Pt-based ORR catalysts, mainly due to the relatively low active site density of NPMCs.^12^ To ensure the overall ORR performance, a common practice is to increase the loading amount or thickness of the NPMC catalyst layer at cathodes, which would inevitably increase the local O_2_ transport resistance due to the elongated diffusion path.^13^ Accordingly, many efforts are devoted to increasing the active site density of NPMCs toward the ORR, which are mainly implemented by engineering the physicochemical structure of NPMCs, such as heteroatom doping,^14,15^ porosity engineering,^12,16^ regulated thermal treatment,^17^ and decoration with a small amount of Pt.^18^

It is documented that the porosity structure of NPMCs, which determines the contents of micropores, mesopores, and macropores, also exerts an effect on the overall ORR performance.^12^ Specifically, micropores accommodate a significant portion of active sites, and macropores facilitate the mass transfer of reactants/products to and from active sites with little resistance throughout the catalyst layer, while mesopores can not only host active sites but also function as the passage between the bulk phase to the catalytic active sites in micropores.^16^ To maximize the efficiency of NPMCs toward the ORR, it is essential to not only synthesize catalytic materials with abundant active sites but also ensure high utilization of these active sites by promoting the mass transfer within the pores.^19,20^ As a matter of fact, many electrocatalysts still suffer from low utilization due to the unoptimized nano-/microinterfaces.^16^ For instance, Strasser et al. performed a comprehensive study on assessing the active site number density of Fe–N–C catalysts using a combined nitrite reduction and CO cryoadsorption approach and confirmed that a significant portion of ORR active sites, especially those in the micropores of Fe–N–C, were not accessible under a conventional electrochemical environment.^21^

In the current study, we demonstrate that incorporation of a small amount of ionic liquid (IL) [BMMIM][NTf_2_] (structure is shown in Figure S1, Supporting Information) can greatly enhance the utilization of the active sites within zeolitic imidazolate framework-derived carbon (ZDC), which recently emerges as a new class of active ORR catalyst.^22^ It is disclosed that both the electrochemically active surface area (ECSA) and the half-wave potential (*E*_1/2_) of the ORR on ZDC depend sensitively on the loading amount of the IL, and a medium IL loading has been identified as optimal. Specifically, the ECSA of ZDC can be enhanced from 52.3 m^2^ g^–1^ on pristine ZDC up to 105.9 m^2^ g^–1^, while the *E*_1/2_ value of the ORR on ZDC is positively shifted by up to 18 mV after the IL modification. Moreover, it is also disclosed that the active sites within the micropores of pristine ZDC are not fully utilized due to incomplete surface wetting, while the added IL would preferentially fill the micropores via the capillary action, which greatly promotes the accessibility of these micropores and thus boosts the ORR performance. These results not only demonstrate a facile approach to improving the ORR performance of NPMCs but also have great implications for designing high-performing catalysts involving interface wetting.

## 2 Experimental Section

### 2.1 Materials

2-Methylimidazole (C_4_H_6_N_2_, 98%), cobalt nitrate hexahydrate [Co(NO_3_)_2_·6H_2_O, 99%], and Nafion solution (5 wt %) were purchased from Aladdin Chemistry Co., Ltd. Ethanol (EtOH) and methanol (MeOH, 99.7%) were purchased from Sinopharm Chemical Reagent Co., Ltd. 1-Butyl-2,3-dimethylimidazolium bis(trifluoromethanesulfonyl)imide ([BMMIM][NTf_2_]) was purchased from Maclin Chemical Inc. 1-Ethyl-2,3-dimethylimidazolium bis(trifluoromethanesulfonyl)imide ([EMMIM][NTf_2_]) and 1-hexyl-2,3-dimethylimidazolium bis(trifluoromethanesulfonyl)imide ([HMMIM][NTf_2_]) were purchased from Shanghai Acmec Biochemical Co., Ltd. All the chemicals and reagents used in this study were of analytical grade and used without further purification. Ultrapure water with a resistivity of 18.2 MΩ cm (@25 °C) from a Hitech water purification system was used in all our experiments.

### 2.2 Synthesis

#### 2.2.1 Synthesis of ZIF-67

ZIF-67 particles were synthesized by modifying a literature protocol,^23^ and the typical synthetic procedure is as follows: 2-methyl imidazole (24 mmol) was first dissolved in 40 mL of a mixed solvent of methanol and ethanol with a volumetric ratio of 1:1, while Co(NO_3_)_2_·6H_2_O (6 mmol) was dissolved in another 40 mL of mixed methanol and ethanol. Both solutions were then mixed together under continuous stirring for 10 s. The resultant purple solution was then left undisturbed overnight at room temperature. The precipitate was collected and washed with ethanol three times by centrifugation, and then dried in a vacuum oven at 80 °C for 24 h to give ZIF-67 particles.

#### 2.2.2 Synthesis of ZIF-derived Carbon

ZDC materials were synthesized through the pyrolysis of the as-synthesized ZIF-67. Specifically, ZIF-67 particles were placed in a ceramic crucible and then loaded in a programmable tube furnace. Under flowing nitrogen (18.7 NL h^–1^), the sample was then heated up to 800 °C at a ramp rate of 200 K h^–1^, and kept for 2 h. Thereafter, the furnace naturally cooled down to room temperature. The black solids were then collected and washed three times using DI water by centrifugation, and vacuum dried at 80 °C overnight to obtain ZDCs.

#### 2.2.3 IL Modification

IL modification to the as-prepared ZDC materials was performed by a simple mixing approach. First, the IL solutions were prepared by dispersing [BMMIM][NTf_2_] in ethanol, and the concentration of IL is 20 vol % (32.2 wt %). IL modification was implemented by mixing a certain amount of the IL solution with the suspension of ZDC, which was prepared by dispersing the solid sample in a mixed solution of deionized water and isopropanol, followed by an intensive sonication treatment. The resultant IL-modified ZDC samples are denoted as ZDC–IL. The mass ratio of IL to ZDC is varied in the range of 0.3 to 6.0.

### 2.3 Instrumentation

High-angle annular dark field-scanning transmission electron microscopy (HAADF-STEM) and energy dispersive spectroscopy (EDS) elemental mapping were carried out on a JEM-2010 microscope (JEOL, 200 kV) equipped with an X-ray detector (X-Max 80, Oxford Instruments). Scanning electron microscopy (SEM) and EDS elemental mapping were performed on an XL-30 FEG microscope (Philips) equipped with an EDAX X-ray detector (CDU Leap XL-30) operated at 30 kV. The N_2_ sorption measurement was performed using a surface area and pore size analyzer (BSD-PM2, Beishide Instrument). The specific surface area of both samples was determined using the Brunauer–Emmett–Teller (BET) equation, while the total pore volume and micropore volume were determined using the Barrett–Joyner–Halenda (BJH) and non-local density functional theory (NLDFT) methods, respectively. Fourier-transform infrared spectroscopy (FTIR) measurements were conducted on a Nicolet IS50 spectrometer (Thermo Scientific). The FTIR spectra were recorded at a resolution of 4 cm^–1^ in the wavenumber range of 500 to 4000 cm^–1^. The sample was grounded with KBr powders and then pressed into a wafer for the FTIR measurements.

### 2.4 Electrochemical Measurements

All electrochemical measurements were performed on a PARSTAT multichannel potentiostat (PMC-1000, AMETEK) at room temperature. A Hg/HgO electrode (RE-1S, ALS) and a graphite rod (PINE) were used as reference and counter electrodes, respectively. A glassy-carbon rotating disk electrode (GC–RDE, 5 mm, PINE) was employed as the working electrode. All electrode potentials reported herein were calibrated against a reversible hydrogen electrode (RHE) using the hydrogen evolution–oxidation reaction on a Pt wire electrode. The RDE was polished to a mirror finish prior to each experiment using an alumina suspension (0.05 μm, BUEHLER), followed by ultrasonically cleaning it in ethanol, acetone, and deionized water, respectively. Catalyst suspensions were prepared by dispersing ZDC powder (5 mg) in the mixture of water (72.5 vol %), isopropanol (22.5 vol %), and 5 wt % Nafion solution (5 vol %). The suspensions were sonicated five times in an ultrasonic homogenizer (UP200ST, Hielscher). A certain amount of the suspension was then drop-casted on the RDE working electrode and dried under an argon flow to give a homogeneous catalyst layer. The catalyst loading on the working electrode was controlled at 255.1 μg cm^–2^.

The ORR measurements were performed in an O_2_-saturated 0.1 M KOH electrolyte. Prior to the ORR measurements, the catalyst-coated RDE working electrode was electrochemically pretreated using potential cycling for 20 cycles in the potential range of 0.25 and 1.2 V vs RHE with a scan rate of 50 mV s^–1^. The linear sweep voltammetry (LSV) curves with a scan rate of 10 mV s^–1^ were then recorded at the RDE rotation rates of 400, 800, 1200, 1600, 2000, and 2500 rpm. LSV curves were then corrected by subtracting the capacitive current, which is determined by recording the corresponding LSV curves in N_2_-saturated 0.1 M KOH electrolyte. The iR-compensation was implemented by determining the solution resistance using electrochemical impedance spectroscopy (EIS) with an AC amplitude of 5 mV, as detailed in our previous work.^24^ The mass transfer corrected kinetic current density (*J*_K_) and electron transfer number during the ORR were determined using the Koutecky–Levich equation
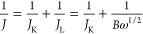

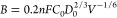
where *J* is the baseline-corrected experimentally measured current density, *J*_K_ and *J*_L_ are the kinetic current density and diffusion limiting current, respectively. ω is the angular velocity (rpm) of the RDE, *n* is the electron transfer number per O_2_ molecule during the ORR, *F* is the Faraday constant (96485.3 C mol^–1^), and *C*_0_ is the saturated concentration of O_2_ in 0.1 M KOH electrolyte.

To determine the ECSA of different samples, CV measurements were conducted in the potential range from −0.6 to −0.2 V vs Hg/HgO, where there is no Faradaic process. The scan rates range from 5 to 100 mV s^–1^. The ECSA can then be calculated according to the following equation
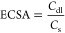
where *C*_dl_ is the double layer capacitance, and *C*_s_ is the capacitance for a standard with 1 cm^2^ of surface area.

The accelerated degradation test (ADT) was performed in O_2_-saturated 0.1 M KOH electrolyte by conducting potential cycling in the range of 0.6 to 1.0 V for up to 2000 cycles. The scan rate was set to 50 mV s^–1^. The ORR polarization curves were recorded after 1000 and 2000 cycles.

## 3 Results

### 3.1 Structural and Morphological Characterizations

**Figure 1:**
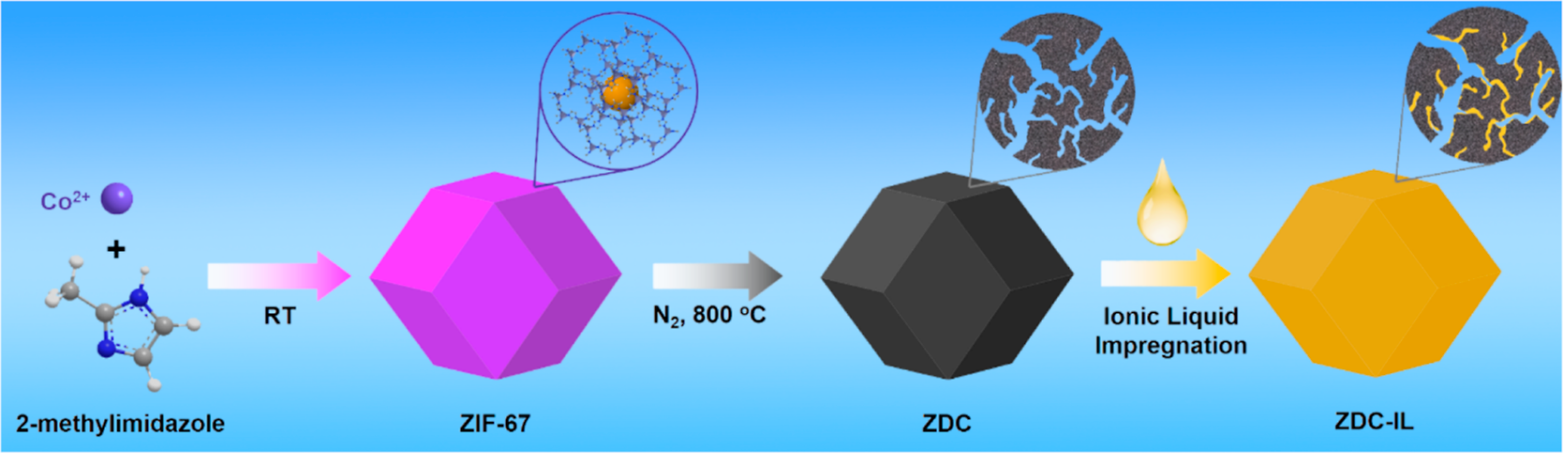
Schematic illustration of the synthesis of pristine and IL-modified ZDC materials.

The synthetic procedure of pristine and IL-modified ZDC is shown schematically in Figure 1. In the current study, monodisperse ZIF-67 nanocrystals were first synthesized using Co^2+^ as the metallic node and 2-methyl imidazole as the linker. Carbonization of the ZIF-67 was carried out through pyrolysis treatment under flowing nitrogen at 800 °C for 2 h, which gives the ZIF-67-derived carbon (ZDC). The crystalline nature of the as-prepared ZDC can be confirmed by powder X-ray diffraction (XRD, Figure S2, Supporting Information). The XRD peak at around 25.8° corresponds to the (002) diffraction of graphitic carbon, and the other peaks at 44.3, 51.6, and 75.9° can be attributed to the trapped cobalt nanoparticles.^25^ The IL-modified ZDC (ZDC–IL) was prepared by adding IL into the ZDC suspension, followed by intensive sonication treatment before preparing the working electrode. Due to the hydrophobic nature of the IL ([BMMIM][NTf_2_]), the IL modification is expected to change the hydrophilic/phobic state of the catalyst.^26^ Accordingly, we carried out water contact angle measurement on the pristine and IL-modified ZDC samples. It turns out that the surface of ZDC can be easily wetted by water (Figure S3, Supporting Information), while in contrast the contact angle of water on ZDC–IL was around 133°. This result confirms that the presence of IL can cause a dramatic change in the ZDC surface from a hydrophilic to a hydrophobic state.

**Figure 2:**
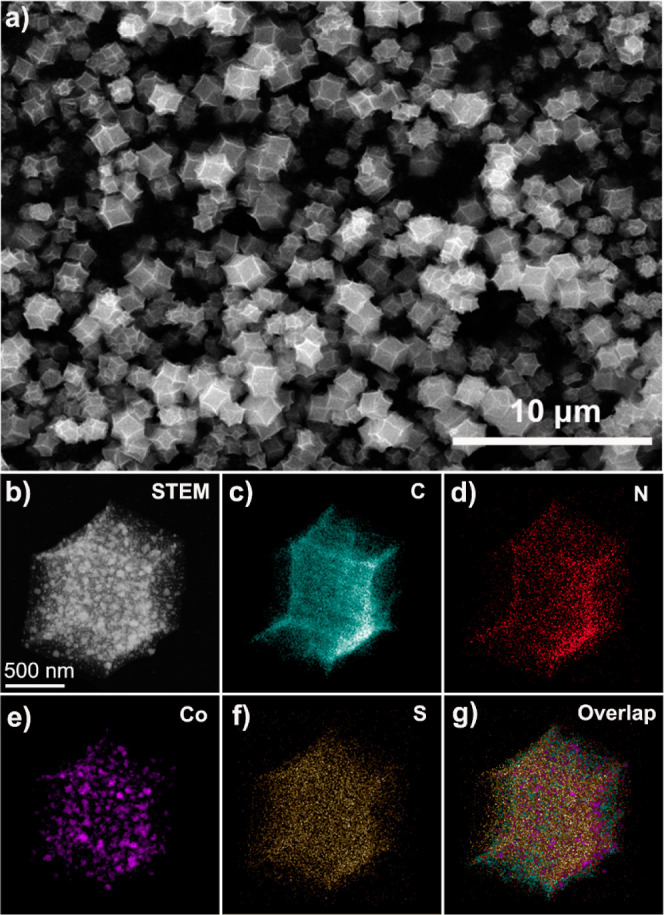
(a) SEM, (b) HAADF–STEM, and (c–g) EDS mapping images of ZDC–IL (mass ratio of IL/ZDC: 1.2).

The size and morphology of the as-prepared ZDC materials before and after the IL modification were characterized by SEM, as shown in Figures 2a and S4 (Supporting Information). The resultant ZDC particles exhibit a characteristic rhombic dodecahedron shape with a typical particle size of 1.2 μm. After loading with IL, no change in either the morphology or particle size can be identified, and the ZDC particles are homogeneously dispersed. HAADF-STEM and EDS elemental mapping techniques were employed to probe the spatial distribution of IL on the ZDC material. As shown in Figures 2b–g, S5 and S6 (Supporting Information), besides the signals of C, N, and Co from the ZDC, characteristic signals of S/F from the IL ([BMMIM][NTf_2_]) can be clearly identified, which are distributed over the ZDC particle without any localized aggregation, implying a homogeneous distribution of IL species.

**Figure 3:**
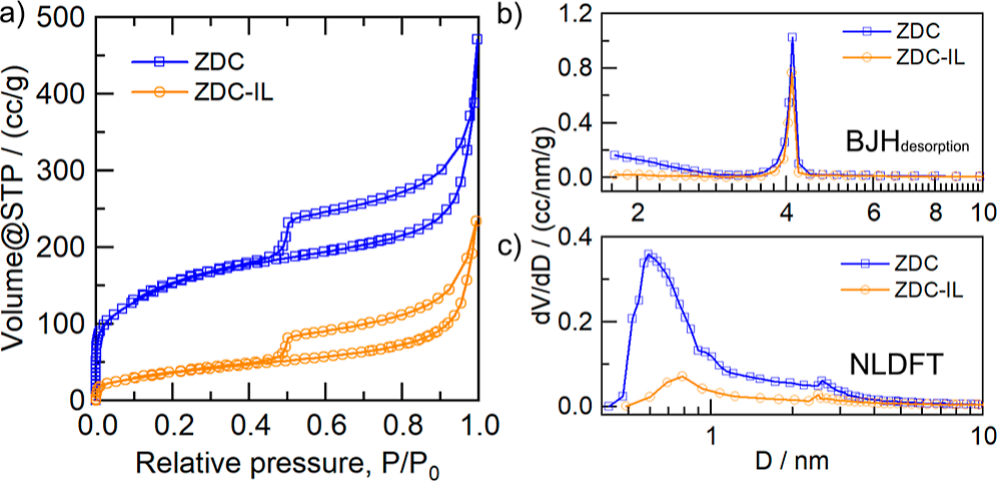
(a) N_2_ adsorption–desorption isotherms at 77.3 K for pristine and IL-modified ZDC materials, and their corresponding PSDs determined by (b) BJH desorption and (c) NLDFT method, respectively.

To gain more insights into the filling behavior of the IL phase within the pores of ZDC materials, we conducted nitrogen sorption analysis to assess the surface area and pore characteristics of ZDC before and after the IL modification. The pristine ZDC exhibits a BET surface area of 548 m^2^ g^–1^ and a total pore volume of 0.61 mL g^–1^. In contrast, ZDC–IL possesses a much smaller surface area (134 m^2^ g^–1^) and total pore volume (0.34 mL g^–1^) due to the pore filling by the IL phase. As shown in Figure 3a, the nitrogen sorption isotherms of both ZDC and ZDC–IL can be identified as a hybrid type of I(b)–II with a type H3 hysteresis, which is analogous to other porous carbon materials with wide pore size distributions (PSD).^27−29^ It is notable that the N_2_ uptake in the micropore region (low relative pressure region) is significantly reduced, as shown in the isotherm curves. Specifically, the micropore volume of ZDC is decreased from 0.24 to 0.04 mL g^–1^, which corresponds to 74% of the reduction in the total pore volume, implying that the IL would first fill the micropores of ZDC. The PSD analyses indicate that ZDC and ZDC–IL possess comparable PSD in the mesopore region (Figure 3b), while the amount of micropores is significantly reduced in the presence of IL (Figure 3c), again confirming that the IL phase tends to preferentially fill the micropores of ZDC. Similarly, Duclaux et al. studied the adsorption of ILs on carbon materials and also found that several imidazolium-based ILs would be preferentially adsorbed in the micropores instead of the mesopores of activated carbons.^30^ Previously, we found that IL ([MTBD][NTf_2_]) would first fill the micropores of carbon-supported Pt nanoparticles and Fe–N–C ORR catalysts based on comprehensive N_2_-sorption analyses.^28,31,32^ This phenomenon is not surprising when considering the inversely proportional relationship between the pore radius and the capillary pressure,^33,34^ which acts as the main driving force for the liquid to fill the pores.

### 3.2 Electrochemical Characterization

The electrochemical properties of pristine and IL-modified ZDC were studied using the thin-film RDE technique. The CV measurements at different scan rates were first performed. The ECSA values can thus be estimated by the double-layer charging capacitance, which is determined by performing CV measurements at different scan rates.^24^ The CV curves of pristine ZDC recorded at scan rates ranging from 5 to 100 mV s^–1^ are shown in Figure S7a. Based on the average capacitance value determined using both the anodic and cathodic scans (Figure S7b), the ECSA of pristine ZDC is determined to be 52.3 m^2^ g^–1^. Figure S7c displays the ORR polarization curves with RDE rotation rates of 400–2500 rpm. The electron transfer number and mass transfer corrected kinetic current can thus be calculated according to the Koutechy–Levich (K–L) equation.^35^ As shown in Figure S7d, the K–L plots show linear dependence behavior between *J*^–1^ and ω^–1/2^ (where *J* is the measured current density and ω is the RDE rotation rate in rpm). The calculated electron transfer number (*n*) is around 4 at 0.8 V, while at a lower electrode potential (0.6 V), the *n* values are slightly decreased to 3.7. Nonetheless, it can be seen that the ORR is taking place predominantly via the 4-electron pathway over the ZDC catalyst.

**Figure 4:**
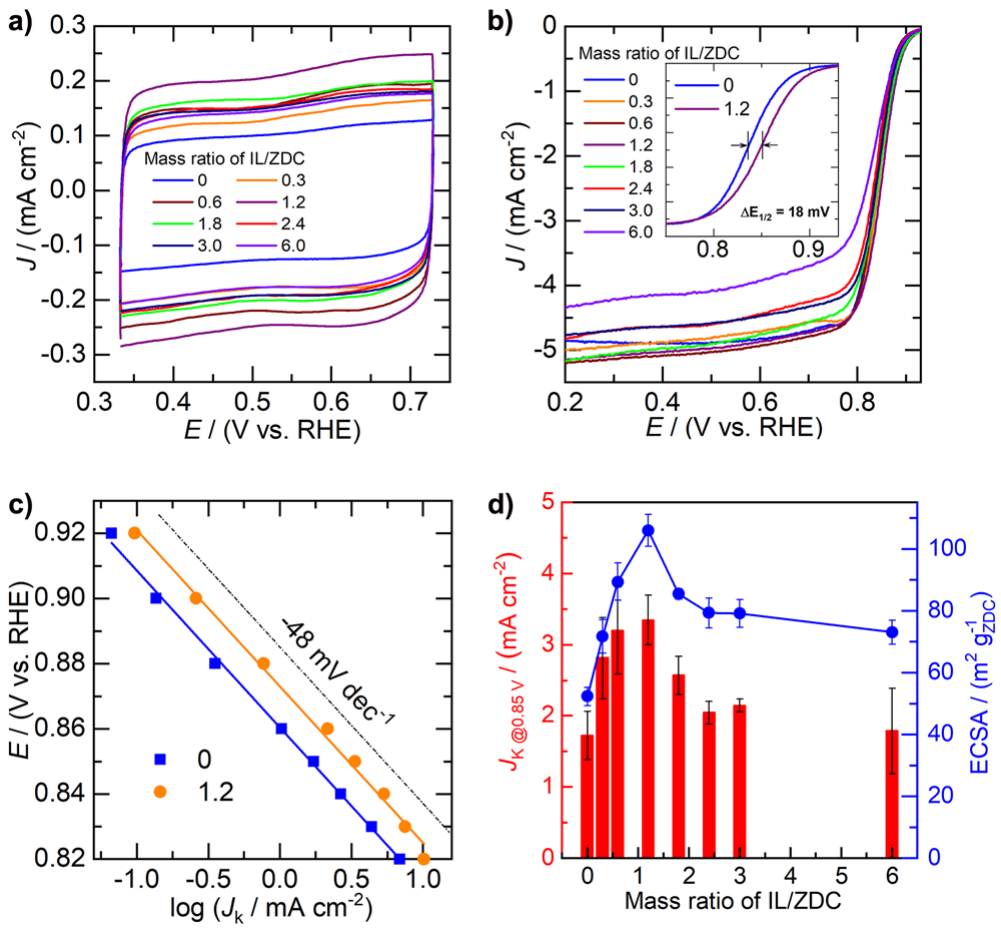
Electrocatalytic properties of ZDC with varied IL loadings. (a) Cyclic voltammetry curves recorded in N_2_-saturated 0.1 M KOH electrolyte with a scan rate of (20 mV s^–1^), (b) ORR polarization curves recorded in O_2_-saturated 0.1 M KOH electrolyte with a scan rate of 10 mV s^–1^, and (c) obtained Tafel plots. (d) Summary of ECAS and ORR kinetic current at 0.85 V for ZDC with varied IL loadings.

Figure 4a shows the CV curves of IL-modified ZDC with various loading amounts of IL (mass ratio of IL to ZDC: 0–6). It can be observed that the presence of IL has strongly influenced the capacitive current (or ECSA) of ZDC. Specifically, the ECSA is increased from 52.3 on pristine ZDC up to 105.9 m^2^ g^–1^ on ZDC–IL-1.2. Interestingly, further increasing the IL loading amount has induced a slight decline in ECSA. The electrocatalytic ORR performance of the pristine and IL-modified ZDC was evaluated at room temperature in an O_2_-saturated KOH solution. The ORR polarization curves recorded in anodic directions are shown in Figure 4b. The diffusion-limiting current densities range from 4.3 to 5.1 mA cm^–2^, which are obtained at potentials below 0.6 V for all the catalysts. Inspection of the ORR polarization curves reveals that the half-wave potential (*E*_1/2_) of ZDC has positively shifted after the IL modification, regardless of the loading amount of IL. The maximum positive shift in *E*_1/2_ value is obtained on the ZDC–IL with a moderate mass ratio of IL/ZDC (i.e., 1.2), which is 18 mV relative to that of the pristine ZDC, as illustrated in the inset of Figure 4b. The Tafel slope analyses indicate that comparable Tafel slope (48 mV s^–1^) observed for pristine and IL-modified ZDC catalysts (Figure 4c), suggesting that the introduction of IL has not altered the reaction kinetics or mechanism of the ORR. To quantify the influence of the loading amounts of IL on ORR activity, the mass transfer corrected kinetic current at 0.85 V (*J*_K@0.85 V_) was calculated and compared in Figure 4d. It can be observed that the ORR activity of ZDC strongly correlates with the loading amount of IL. A distinct dependence of *J*_K@0.85 V_ on the mass ratio of IL/ZDC is identified, with the maximum value of 3.3 mA cm^–2^ obtained on ZDC–IL-1.2, which is almost two times that of pristine ZDC (1.7 mA cm^–2^). Interestingly, similar dependence behavior of ECSA on the mass ratio of IL/ZDC can be observed. These results corroborate that the boosted ORR activity on the IL-modified ZDC would benefit largely from the enhanced ECSA.

The influence of IL modification on the electrochemical durability of ZDC was also evaluated by performing ADT, which was implemented by repetitive voltammetric cycling in the potential range of 0.6 to 1.0 V. As shown in Figure S8, the polarization curve of the IL-modified ZDC (ZDC–IL-1.2) was negatively shifted by less than 4 mV, while the ECSA was almost fully retained after the ADT. Both the ORR activity and ECSA of ZDC–IL-1.2 after the ADT are still substantially superior to those of the initial pristine ZDC. Attempt was also made to characterize the ZDC–IL-1.2 after the above electrochemical tests using FTIR, and it turns out that the characteristic vibrational features of the IL can still be clearly distinguished (Figure S9). These results provide a solid piece of evidence that there is no severe loss of IL during the electrochemical tests, and the IL boosting effect can be well maintained, which is crucial for the practical application of IL-modified electrocatalysts.

## 4 Discussion

The aforementioned results clearly demonstrate that the ORR activity of ZDC materials can be easily boosted by introducing IL, and both the kinetic current density and ECSA of ZDC exhibit a unique dependence on the loading amount of IL, as shown in Figure 4d. It is intriguing to observe two regimes when the mass ratio of IL/ZDC is increased. The first regime is from 0.6 to 1.2, where both the ECSA and ORR activity keep increasing with the IL loading amount. The second regime is from 1.2 to 6.0, where both the ECSA and ORR activity first decline and then get leveled off. These results imply a profound change in the role of the IL on influencing the electrochemical properties of ZDC when the mass ratio of IL/ZDC increases from 1.2 to 1.8.

There have already been many studies concerning the boosting effect of ILs on the ORR activity of both the Pt-based catalysts and NPMCs.^32,36−38^ Nevertheless, a consensus conclusion or an exclusive mechanistic understanding about the origin of the IL boosting effect remains elusive. Erlebacher et al. proposed in their pioneering work that the boosted ORR kinetics on IL-impregnated PtNi film would mainly stem from the higher O_2_ solubility in IL than that in aqueous solution.^39^ To clarify the influence of O_2_ solubility in ILs on the ORR kinetics, Etzold et al. investigated the ORR performance of Pt/C catalysts modified with two ILs ([C_4_C_1_im][NTf_2_] and [C_4_C_1_im][BETI]) featuring different O_2_ solubility.^26^ It turns out that both ILs can boost the ORR activity of Pt/C by a factor of two to three, while the influence of the IL identity (or O_2_ solubility in the ILs) is not pronounced.^40−43^ Since anions of ILs play a decisive role in determining O_2_ solubility in ILs, Huang et al. studied the influence of O_2_ solubility on the ORR activity by modifying Pt/C catalysts using ILs with various anions.^44^ They suggested that the beneficial role of the high O_2_ solubility in ILs has been largely counteracted by the slow O_2_ diffusion through the ILs and the long mean free path within the ILs. A recent work also reported no direct correlation between the O_2_ solubility and ORR activity of Pt.^45^ Based on these results, it can be seen that the IL-boosting effect is less likely to (solely) originate from the high O_2_ solubility in ILs.

**Figure 5:**
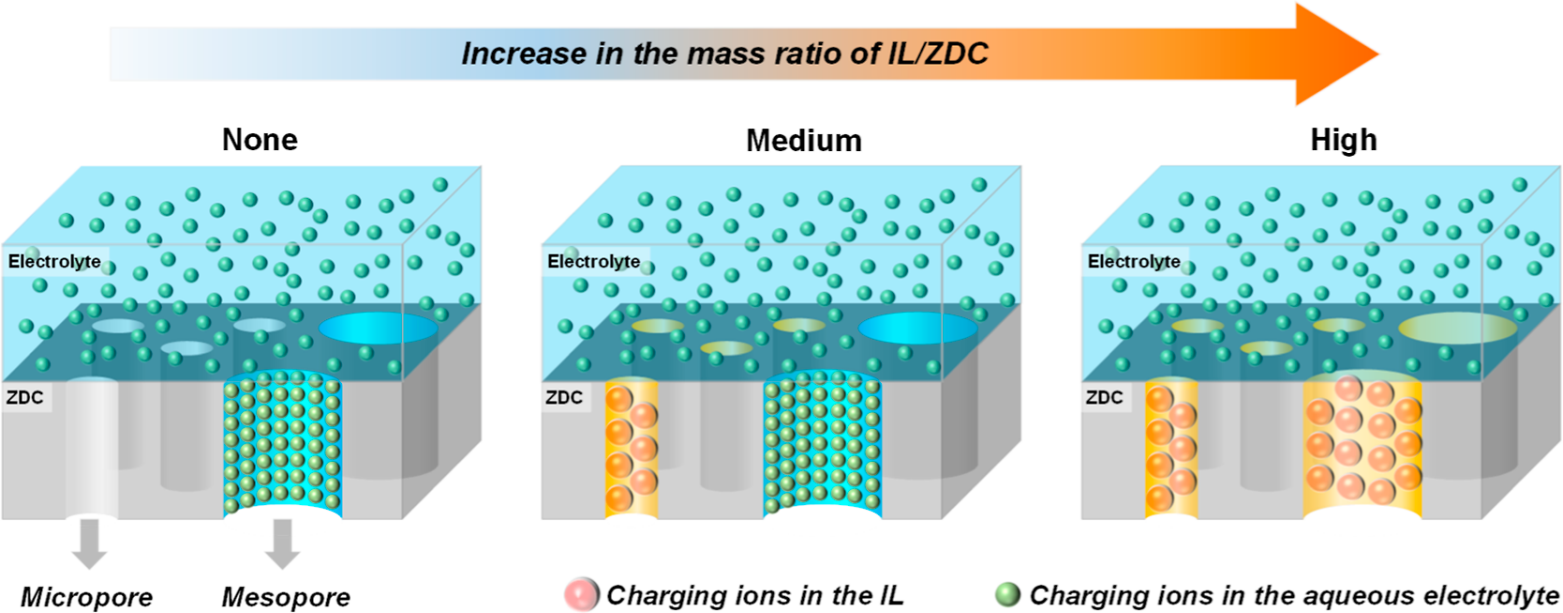
Schematic illustration for the formation of an electrochemical double layer on ZDC with no, medium, and high IL loadings.

As shown in Figure 5a, the introduction of the IL has greatly enhanced the capacitance current of ZDC, which implies that the presence of IL can make the surface of ZDC more accessible to the electrolyte solution, as reflected by the enhanced ECSA value of ZDC after IL modification. Moreover, the good correlation between the ECSA and the ORR activity in terms of kinetic current density leads us to hypothesize that the IL-boosted ORR activity would benefit largely from the enhanced accessible surface area of ZDC after the IL modification. Nevertheless, this hypothesis is actually counterintuitive because for a given carbon material its capacitance is expected to be lower in ILs than that in aqueous electrolytes.^46^ Thus, it is critical to understand how the presence of the IL can increase the accessible surface area of ZDC materials.

Balke et al. pointed out that insertion of ILs into the pores of carbon materials resulted in pore swelling, i.e., pore volume expansion.^47^ Therefore, an intuitive rationale for the enhanced ECSA of ZDC in the presence of IL would lie in the pore volume expansion effect. Nonetheless, molecular dynamics simulations indicate that filling the pores of carbon materials with the IL [C_2_C_1_im][NTf_2_] can only lead to a pore volume expansion by 6% (∼3% in surface area),^47^ which is thus less likely to be responsible for the 2× enhancement in ECSA of ZDC–IL.

In the current study, the ECSA values were estimated based on the capacitance current densities. The principle for this methodology is that the solid electrocatalysts can act as capacitors and build-up charges at the interfaces between the solid electrocatalysts and liquid electrolytes. It can be seen that the capacitance current can relate to both the accessible (or exposed) surface of solid electrocatalysts and the identity of the charging ions. Herein, the presence of the IL phase at the electrochemical interfaces can alter both of the above parameters.

Regarding the accessible surface area, besides the inherent surface area, it is also significantly influenced by the wetting behavior of the solid electrocatalysts,^33,48,49^ which is usually determined by the complex solid–liquid interfacial interactions mainly including electrostatic and van der Waals forces.^50−52^ It is well documented that ILs can alter the microenvironment at the electrochemical interfaces, and thus in principle, they are also expected to impose a profound impact on whether the surface of solid electrocatalysts could be fully accessible to the electrolyte solution. By revising the N_2_-sorption isotherms and PSD curves of ZDC materials with/without the IL (Figure 3), it can be seen that the IL tends to preferentially fill the micropores of ZDC materials. These results make us tend to believe that a significant portion of the micropores of ZDC cannot be easily wetted by the aqueous electrolyte. This may stem from the hydrophobic nature of carbon-based materials, which leads to the formation of the so-called Cassie–Baxter structure containing trapped air within the micropores.^33^ The resultant “gas pockets” within the electrocatalysts prevent the direct contact between the inner pore surface and liquid electrolyte, thus resulting in a compromised ECSA of ZDC. After introducing the IL into the solid catalysts, the IL phase would fill those micropores of ZDC, thus avoiding the formation of those gas pockets and at the same time making the micropores of ZDC accessible to charging ions, as shown schematically in Figure 5. These results lead us to hypothesize that: (1) the conventional carbon-based materials may not be fully utilized when being employed as electrocatalysts due to the incomplete surface wetting. (2) The IL phase would spontaneously fill the micropores of ZDC due to the capillary force, which greatly enhance the utilization of ZDC. The comparable Tafel slopes of ORR on the pristine and IL-modified ZDC also implies that the presence of IL has not altered the reaction mechanism or introduced new active species but rather make those active sites within micropores can participate in the reaction. However, when the IL loading is too high, not only the micropores but also the mesopores would be fully flooded by the IL, resulting in a lower capacitance current, likely due to the larger molecular size of the IL than the charging ions in the aqueous solution.^46^ These results demonstrate that introducing a suitable amount of IL can help enhance the utilization of the micropores that are not easily accessible to aqueous electrolyte due to the incomplete surface wetting of ZDC. Nevertheless, introducing too much IL can lead to lower capacitance current due to its bulky size (Figure 5) and, at the same time, cause too much mass transfer resistance for the reactant molecules O_2_, thus leading to lower overall ORR activity.

Moreover, ILs feature great structural variety and flexibility, which allow the design and synthesis of ILs with various structural moieties. There is some recent progress being made in correlating the molecular structure of ILs with the electrocatalytic properties of Pt and NPMCs toward the ORR.^36,41,44,53−55^ These inspiring studies would provide the basis of rationally designing task-specific ILs with desired functionalities, the success of which can help fully leverage the IL modification strategy to construct high-performance electrocatalysts. To this end, herein we made attempt to study the influence of the molecular structure of ILs on the ORR activity of ZDC materials. Specifically, ZDCs were modified with imidazolium-based ILs featuring different cationic chain lengths (Figure S1), i.e., 1-ethyl-2,3-dimethylimidazolium bis(trifluoromethylsulfonyl)imide ([EMMIM][NTf_2_]) and 1-hexyl-2,3-dimethylimidazolium bis(trifluoromethylsulfonyl)imide ([HMMIM][NTf_2_]) in addition to [BMMIM][NTf_2_]. It was found that the ECSA values of the IL-modified ZDCs were significantly higher than those of the pristine ZDC, with a slight decrease observed with the elongation of the cationic chain (Figure S10). Additionally, we observed a strong correlation between the cationic chain length and ORR activity, with the maximum activity (*J*_k@0.85 V_ = 4.1 mA cm^–2^) obtained on ZDC–[EMMIM][NTf_2_]. This result suggests that in addition to enhancing the utilization of active sites within the micropores of ZDC, the IL also plays an important role in controlling the overall reaction rate during the ORR, which can be optimized by rationally engineering the molecular structure of ILs. These intriguing results inspired us to revisit the role of ILs during the ORR.

It is widely known that water plays a crucial role in the ORR process. In the case of the ORR in an alkaline electrolyte, water is a major reactant besides O_2_ molecules. Despite the hydrophobic nature of the ILs used in this study, they exhibit pronounced solubility with water. According to both theoretical predictions and experimental results, ILs with longer cationic chains generally have lower water content.^56,57^ This can be explained by the fact that the longer nonpolar cationic tail of the IL would create a larger structural mismatch with water molecules upon interaction, resulting in a higher misfit energy and ultimately lower water solubility. Therefore, the variation in ORR activity observed in this study as a function of the cationic chain lengths of the ILs may be attributed to the differences in their water content. Based on these results, we can draw a more comprehensive picture about the role of ILs during the ORR. The introduction of a small amount of ILs has selectively filled the micropores of ZDCs, as demonstrated by N_2_-sorption analyses (Figure 3). The IL phase first functions by wetting the micropores initially inaccessible to aqueous electrolyte, thereby improving the utilization of active sites within these regions. Meanwhile, the presence of water in ILs can promote the transportation of H_2_O/OH^–^ within the micropores, which is crucial for the ORR in alkaline electrolyte. In principle, ILs with shorter cationic chain lengths would be more favorable for the migration of water molecules and the shuffling of hydroxide ions through the interconnected water network due to their higher water content.^58,59^ Therefore, in this study, the IL with the shortest cationic chain length (i.e., [EMMIM][NTf_2_]) can exhibit the most pronounced effect in enhancing the ORR performance. We believe that these results provide a refreshing perspective on understanding the role of ILs in affecting the electrocatalytic properties of this emerging system, while we also acknowledge that further extensive efforts, particularly those based on microscopic, spectroscopic, or theoretical studies, are still required to gain more insights into the explicit role of ILs and their synergy with the solid electrocatalysts.

## 5 Conclusions

In conclusion, we demonstrate that the active sites within the micropores of pristine ZDC would not be fully utilized due to the incomplete surface wetting, the influence of which has been largely overlooked in this field. Herein, we disclose that the utilization of ZDC can be dramatically enhanced by incorporating a subtle amount of IL. Specifically, both the ECSA and the ORR activity in terms of kinetic current at 0.85 V can be easily boosted by a factor of 2 after introducing the IL with an optimized loading amount. It is also disclosed that the IL would preferentially fill those micropores of ZDC, thus increasing the accessibility of the active sites within the micropores, while the dissolved water in the IL can promote the transportation of H_2_O/OH^–^ within the micropores. Nevertheless, the drawbacks of the IL such as high viscosity and retarded diffusion of reactant in IL would set in when the loading amount of the IL is too high, which compromises the IL boosting effect toward the ORR. These results pave a new facile way for improving the ORR performance of NPMCs in addition to resorting to complex structure engineering. Considering the great variety of both ILs and emerging NPMCs, it is ensured that there is still much room left for further improving the performance of NPMCs by rationally optimizing the micro-/nanointerfacial structures using ILs.

## Supporting Information Available

Additional information about IL structures, XRD patterns of ZDC, contact angle measurements, SEM/STEM and EDS mapping images, CV curves, K–L plots, and LSV curves at different RDE rotation rates (PDF)
