# Supplementary material for: Increasing Accessible Active Site Density of Non-Precious Metal Oxygen Reduction Reaction Catalysts through Ionic Liquid Modification
Source: ACS Appl Mater Interfaces. 2023 Apr 6;15(15):18781–9. doi: 10.1021/acsami.2c21441 (PMC10119856; doi:10.1021/acsami.2c21441)
Supplement: Supplementary file 1 — am2c21441_si_001.pdf [file am2c21441_si_001.pdf]

Supporting Information

Increasing Accessible Active Site Density of Non-Precious Metal ORR Catalysts Through Ionic Liquid Modification

Gui-Rong Zhang [a,b,*], *Cong Yong* [a], *Liu-Liu Shen* [a,b,c], *Hui Yu* [a], *Kai Brunnengräber* [b], *Timo Imhof* [b], *Donghai Mei* [a,d,*], *and Bastian J.M. Etzold* [b*]

[a] School of Chemical Engineering and Technology, Tiangong University, Binshuixi Road 399, Tianjin 300387, China

[b] Technical University of Darmstadt, Department of Chemistry, Ernst-Berl-Institut für Technische und Makromolekulare Chemie, Alarich-Weiss-Str. 8, 64287 Darmstadt, Germany

[c] School of Chemistry, Tiangong University, Binshuixi Road 399, Tianjin 300387, China

[d] School of Environmental Science and Engineering, Tiangong University, Binshuixi Road 399, Tianjin 300387, China

E-mails: grzhang@tiangong.edu.cn; dhmei@tiangong.edu.cn; etzold@tu-darmstadt.de

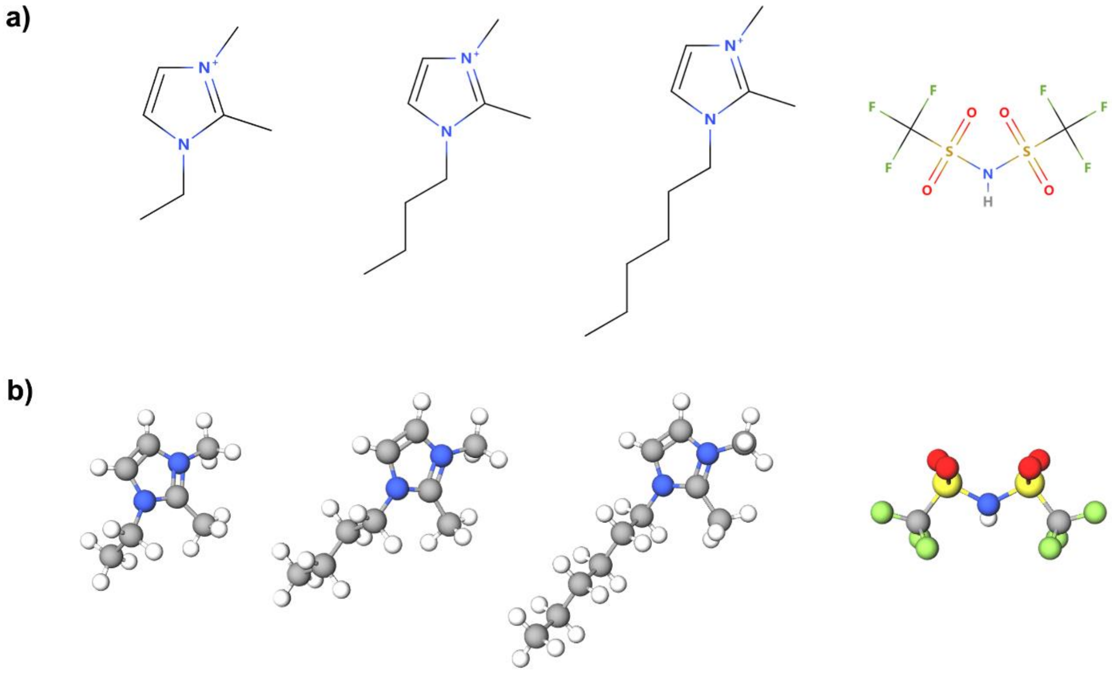

Figure S1. (a) Structure formula and (b) 3D model structure of [EMMIM]$^+$, [BMMIM]$^+$, [HMMIM]$^+$and [NTF$_2$]$^-$.

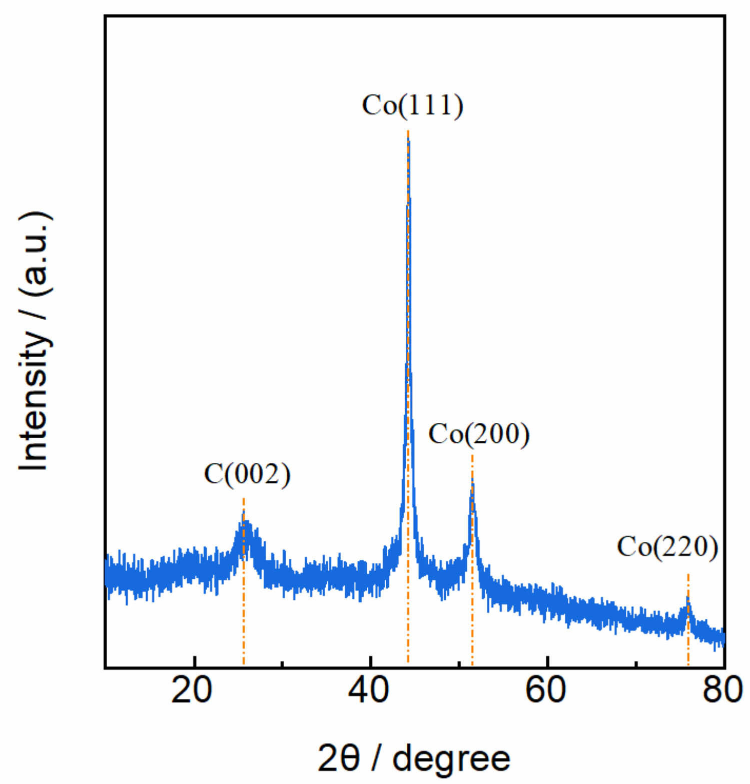

Figure S2. XRD pattern of pristine ZDC material.

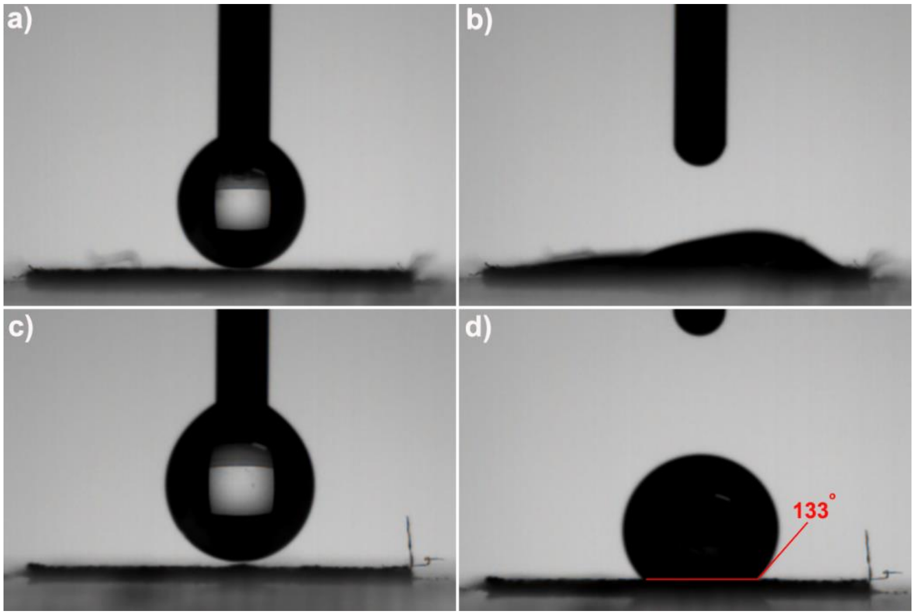

Figure S3. Static water contact angle measurements on a,b) pristine and c,d) IL modified ZDC.

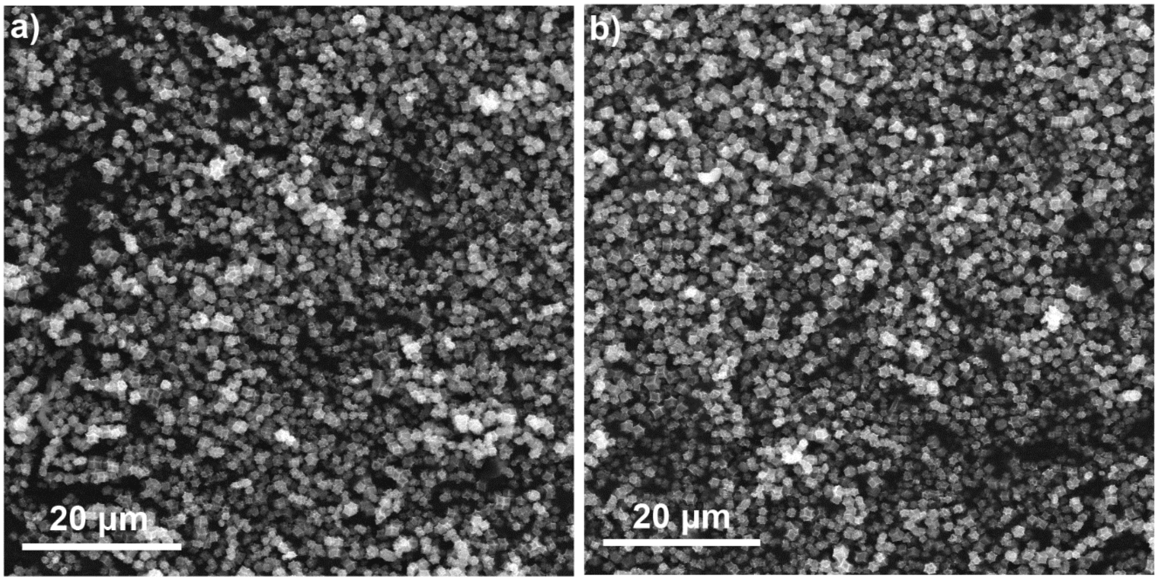

Figure S4. Low magnification SEM images of pristine and IL modified ZDC materials.

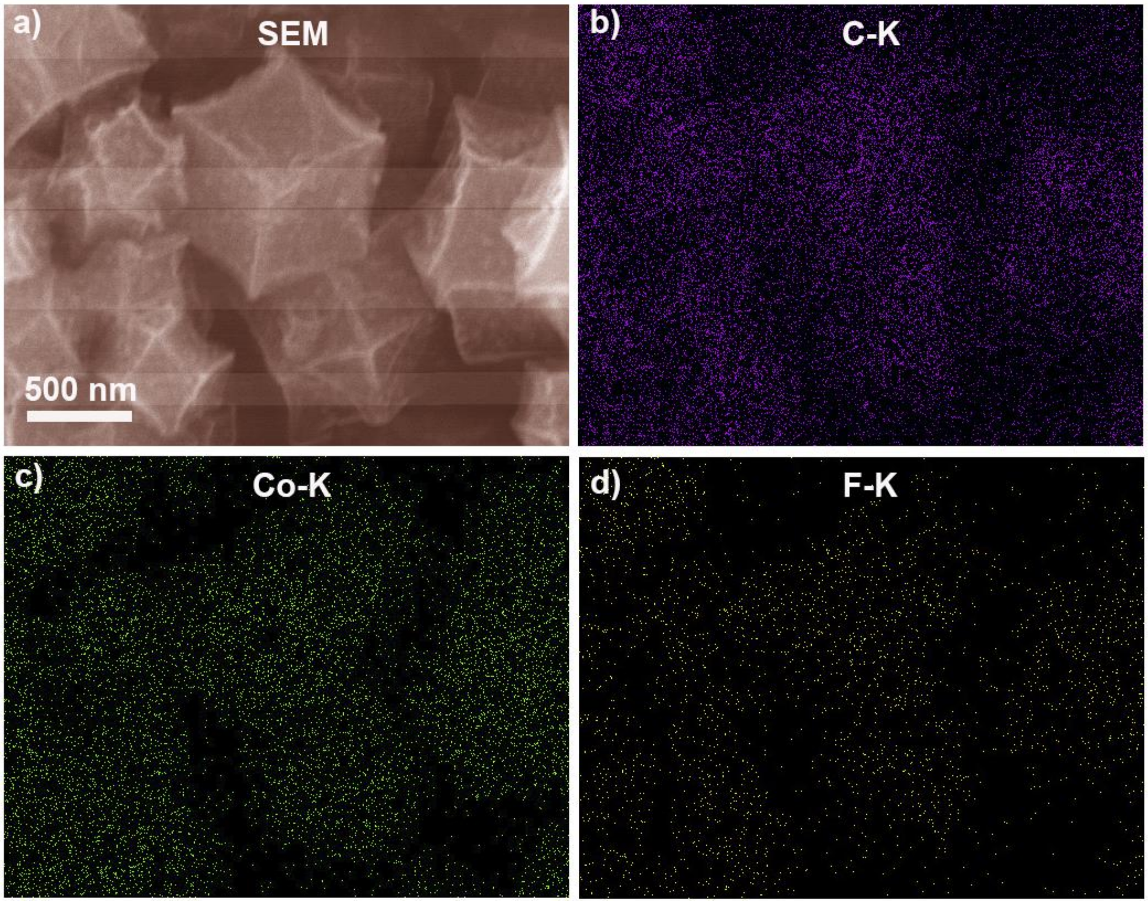

Figure S5. SEM and EDS mapping images of IL modified ZDC.

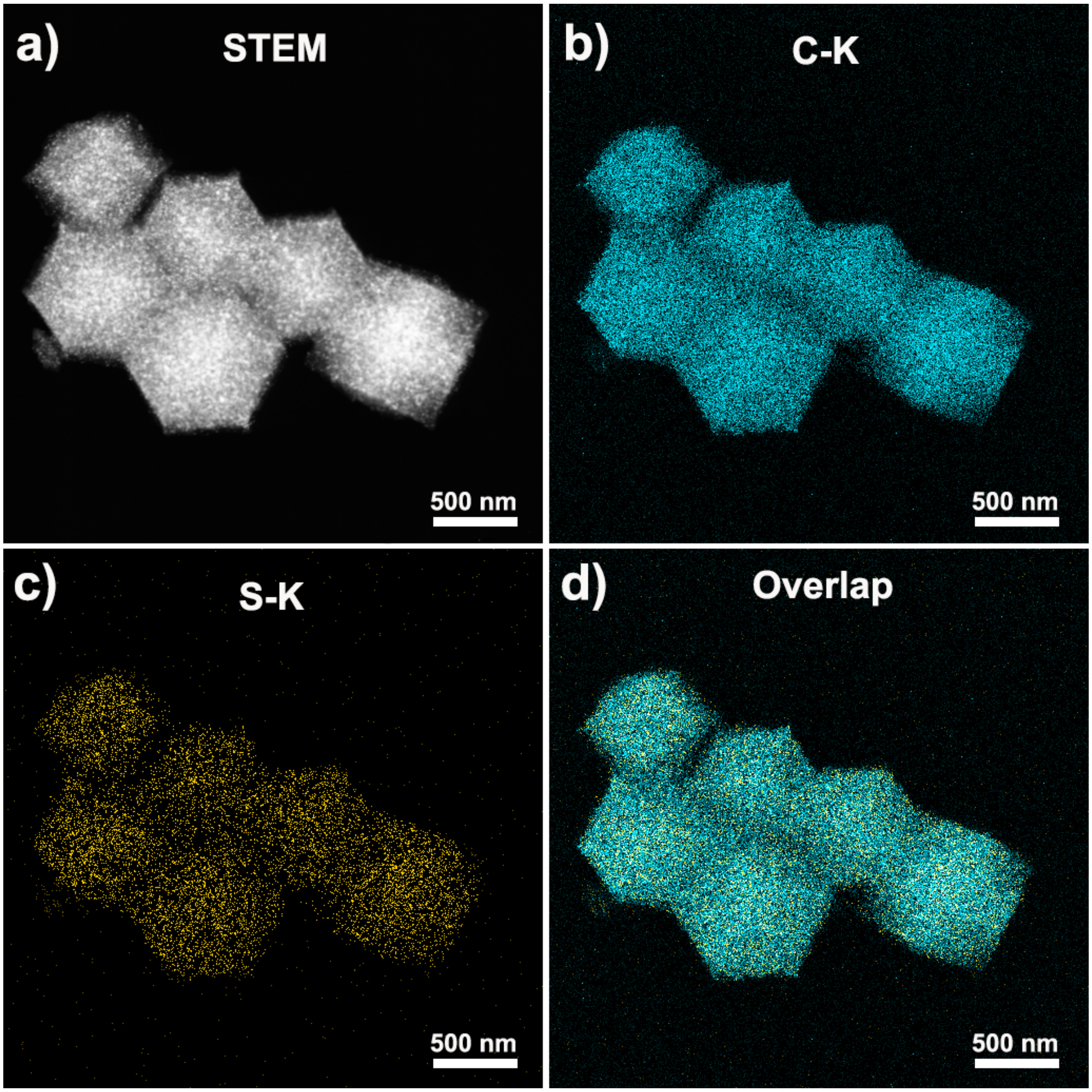

Figure S6. STEM and EDS mapping images of IL modified ZDC.

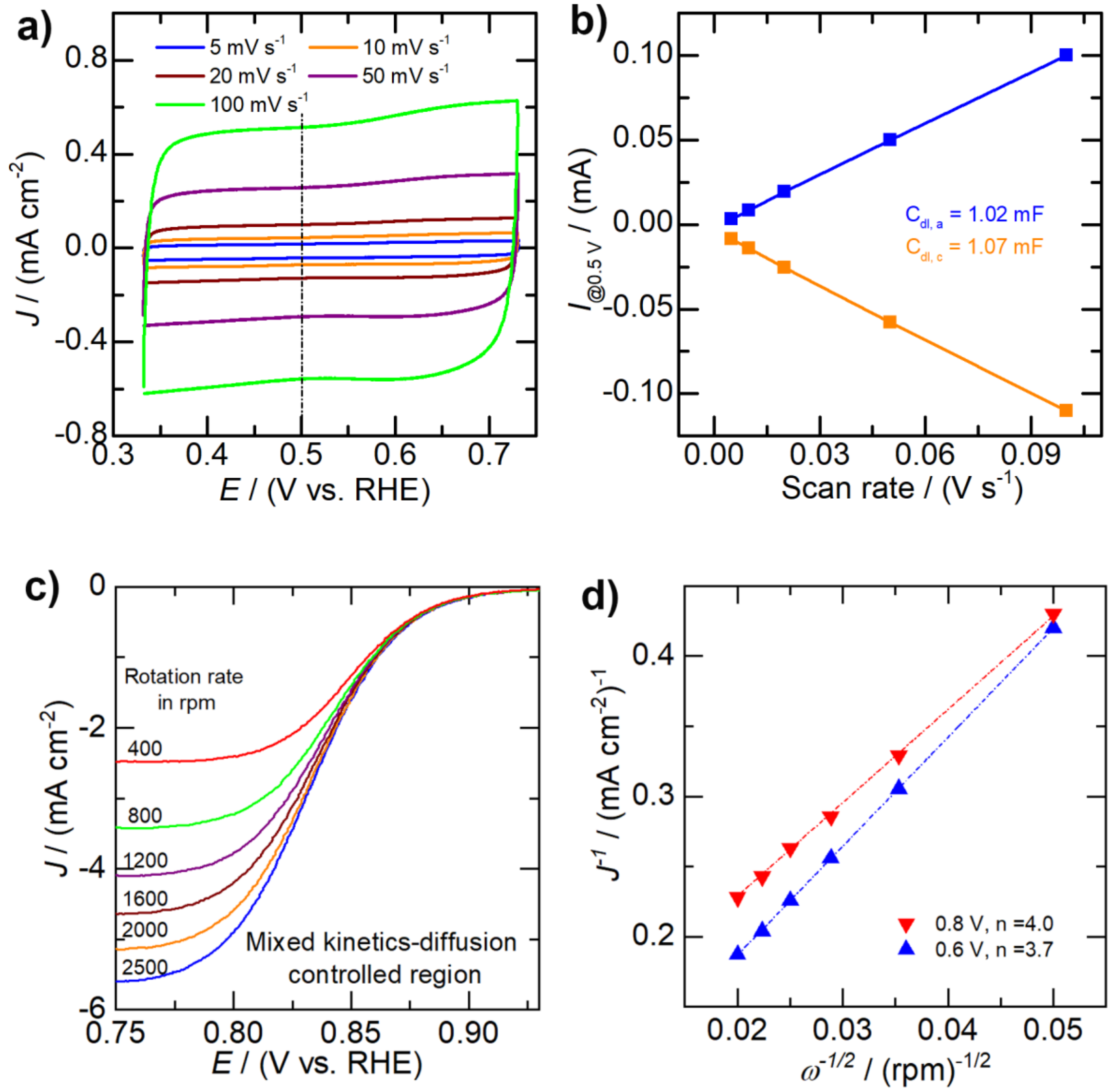

Figure S7. (a) Cyclic voltammetry curves recorded at different scan rates from 5 to 100 mV s^{-1} in N_2-saturated 0.1 M KOH electrolyte; (b) the anodic and cathodic current values extracted at 0.5 V from CV measurements; (c) ORR polarization curves with different RDE rotation rates from 400 to 2500 rpm in O_2-saturated 0.1 M KOH electrolyte; and (d) Koutecky-Levich plots of the pristine ZDC catalyst at different potentials, where the electron transfer numbers are also displayed.

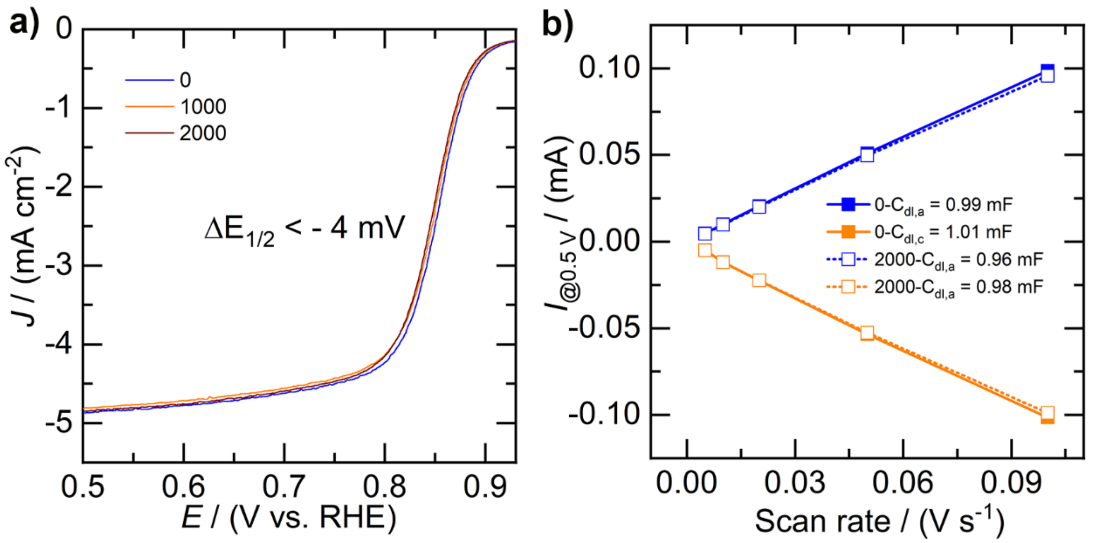

Figure S8. (a) polarization curves for ORR on ZDC-IL-1.2 after 0, 1000 and 2000 cycles in O_2-saturated 0.1 M KOH electrolyte. (b) the anodic and cathodic current values extracted at 0.5 V from the CV measurements after 0 and 2000 potential cycles.

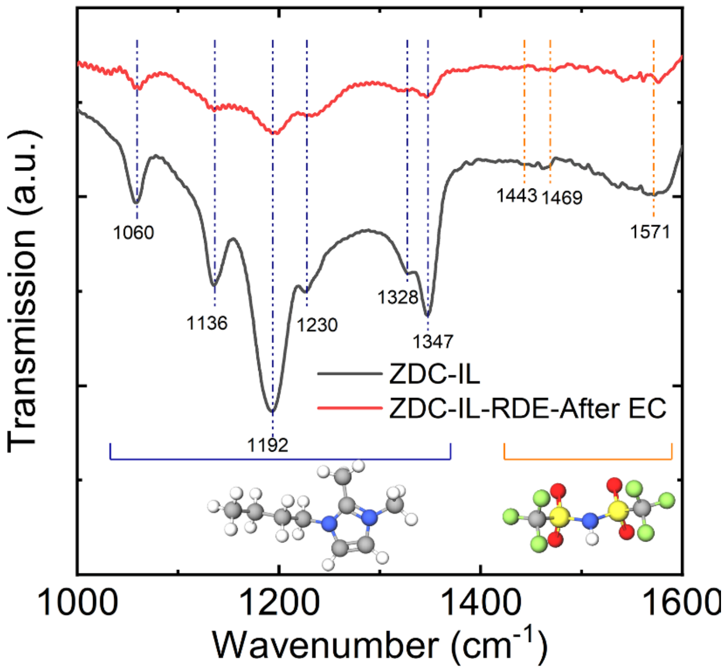

Figure S9. FTIR spectra for ZDC-IL-1.2 powder and ZDC-IL-1.2 after being loaded onto RDE and subjected to electrochemial (EC) measurements. The insets show the structure of cation and anion, respectively.

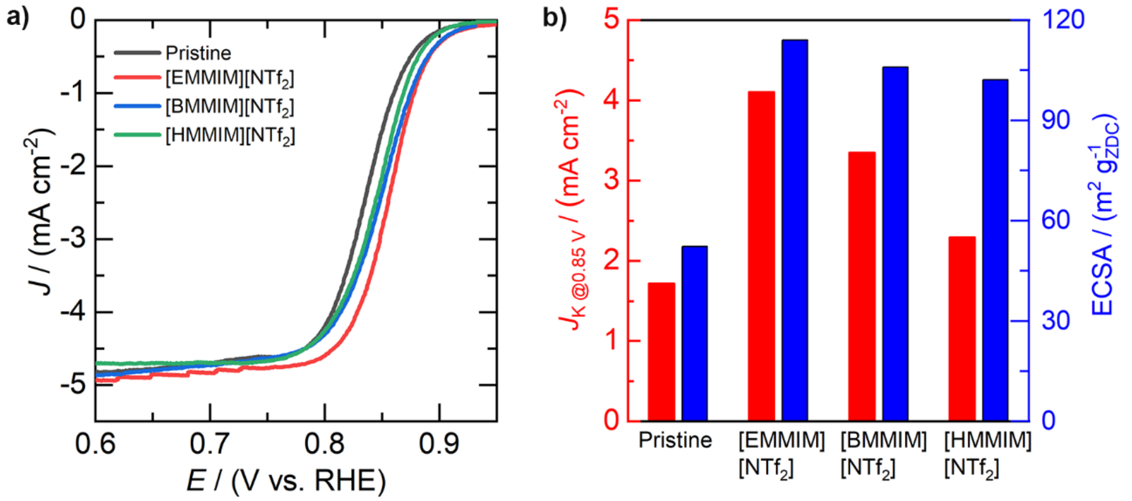

Figure S10. (a) ORR polarization curves recorded in O_2-saturated 0.1 M KOH electrolyte with scan rate of 10 mV s^{-1}, and (b) summary of ECAS and ORR kinetic current at 0.85 V for ZDC modified with different ILs. The mass ratios of IL/ZDC were controlled at 1.2.
